# Neoadjuvant cisplatin and fluorouracil versus epirubicin, cisplatin, and capecitabine followed by resection in patients with oesophageal adenocarcinoma (UK MRC OE05): an open-label, randomised phase 3 trial

**DOI:** 10.1016/S1470-2045(17)30447-3

**Published:** 2017-09

**Authors:** Derek Alderson, David Cunningham, Matthew Nankivell, Jane M Blazeby, S Michael Griffin, Adrian Crellin, Heike I Grabsch, Rupert Langer, Susan Pritchard, Alicia Okines, Richard Krysztopik, Fareeda Coxon, Joyce Thompson, Stephen Falk, Clare Robb, Sally Stenning, Ruth E Langley

**Affiliations:** a Queen Elizabeth Hospital, Birmingham, UK; b The Royal Marsden NHS Foundation Trust, London, UK; c Medical Research Council Clinical Trials Unit at UCL, London, UK; d Centre for Surgical Research, University of Bristol, Bristol and University Hospitals Bristol NHS Foundation Trust, Bristol, UK; e The Northern Oesophago-Gastric Cancer Unit, Royal Victoria Infirmary, Newcastle Upon Tyne, UK; f Leeds Teaching Hospital NHS Trust, Leeds, UK; g Leeds Institute of Cancer and Pathology, University of Leeds, Leeds, UK; h Department of Pathology, GROW School for Oncology and Developmental Biology, Maastricht University Medical Center, Maastricht, Netherlands; i University of Bern, Bern, Germany; j University Hospital South Manchester, Manchester, UK; k Royal United Hospital, Bath, UK; l Birmingham Heartlands Hospital, Birmingham, UK; m Bristol and University Hospitals Bristol NHS Foundation Trust, Bristol, UK; n St George's, University of London, London, UK

## Abstract

**Background:**

Neoadjuvant chemotherapy before surgery improves survival compared with surgery alone for patients with oesophageal cancer. The OE05 trial assessed whether increasing the duration and intensity of neoadjuvant chemotherapy further improved survival compared with the current standard regimen.

**Methods:**

OE05 was an open-label, phase 3, randomised clinical trial. Patients with surgically resectable oesophageal adenocarcinoma classified as stage cT1N1, cT2N1, cT3N0/N1, or cT4N0/N1 were recruited from 72 UK hospitals. Eligibility criteria included WHO performance status 0 or 1, adequate respiratory, cardiac, and liver function, white blood cell count at least 3 × 10^9^ cells per L, platelet count at least 100 × 10^9^ platelets per L, and a glomerular filtration rate at least 60 mL/min. Participants were randomly allocated (1:1) using a computerised minimisation program with a random element and stratified by centre and tumour stage, to receive two cycles of cisplatin and fluorouracil (CF; two 3-weekly cycles of cisplatin [80 mg/m^2^ intravenously on day 1] and fluorouracil [1 g/m^2^ per day intravenously on days 1–4]) or four cycles of epirubicin, cisplatin, and capecitabine (ECX; four 3-weekly cycles of epirubicin [50 mg/m^2^] and cisplatin [60 mg/m^2^] intravenously on day 1, and capecitabine [1250 mg/m^2^] daily throughout the four cycles) before surgery, stratified according to centre and clinical disease stage. Neither patients nor study staff were masked to treatment allocation. Two-phase oesophagectomy with two-field (abdomen and thorax) lymphadenectomy was done within 4–6 weeks of completion of chemotherapy. The primary outcome measure was overall survival, and primary and safety analyses were done in the intention-to-treat population. This trial is registered with the ISRCTN registry (number 01852072) and ClinicalTrials.gov (NCT00041262), and is completed.

**Findings:**

Between Jan 13, 2005, and Oct 31, 2011, 897 patients were recruited and 451 were assigned to the CF group and 446 to the ECX group. By Nov 14, 2016, 327 (73%) of 451 patients in the CF group and 302 (68%) of 446 in the ECX group had died. Median survival was 23·4 months (95% CI 20·6–26·3) with CF and 26·1 months (22·5–29·7) with ECX (hazard ratio 0·90 (95% CI 0·77–1·05, p=0·19). No unexpected chemotherapy toxicity was seen, and neutropenia was the most commonly reported event (grade 3 or 4 neutropenia: 74 [17%] of 446 patients in the CF group *vs* 101 [23%] of 441 people in the ECX group). The proportions of patients with postoperative complications (224 [56%] of 398 people for whom data were available in the CF group and 233 [62%] of 374 in the ECX group; p=0·089) were similar between the two groups. One patient in the ECX group died of suspected treatment-related neutropenic sepsis.

**Interpretation:**

Four cycles of neoadjuvant ECX compared with two cycles of CF did not increase survival, and cannot be considered standard of care. Our study involved a large number of centres and detailed protocol with comprehensive prospective assessment of health-related quality of life in a patient population confined to people with adenocarcinomas of the oesophagus and gastro-oesophageal junction (Siewert types 1 and 2). Alternative chemotherapy regimens and neoadjuvant chemoradiation are being investigated to improve outcomes for patients with oesophageal carcinoma.

**Funding:**

Cancer Research UK and Medical Research Council Clinical Trials Unit at University College London.

## Introduction

Meta-analyses of randomised trials support the use of neoadjuvant chemotherapy or chemoradiotherapy before surgical resection of locally advanced oesophageal cancer to improve survival.^1^, ^2^ Older clinical studies were dominated by squamous cell carcinoma^3^, ^4^, ^5^, ^6^, ^7^, ^8^, ^9^, ^10^, ^11^, ^12^ but the increasing incidence of oesophageal adenocarcinoma, particularly in high-income countries in the past 30 years, indicates a changing epidemiology. Most trials recruited patients with either squamous cell carcinoma or adenocarcinoma^13^, ^14^, ^15^, ^16^, ^17^, ^18^, ^19^, ^20^ and although they were unable to show a survival difference between these two histological tumour types, none of the studies were specifically powered to detect any such difference. Few studies have been done only in patients with oesophageal adenocarcinoma.^21^, ^22^, ^23^, ^24^

**Research in context**

**Evidence before this study**

On Jan 12 and 13, 2017, we searched PubMed and the abstracts of major conferences such as the American Society of Clinical Oncology for publications in English, with no publication date restrictions. The search terms used were “chemotherapy”, “neo-adjuvant (or neoadjuvant)”, and “oesophageal (or esophageal)”. We identified two meta-analyses comparing neoadjuvant chemotherapy with surgery alone in patients with oesophageal cancer. These meta-analyses showed a significant survival benefit after treatment with neoadjuvant chemotherapy, both overall and for the subset of patients with adenocarcinoma who were considered in this study. These meta-analyses were unable to assess the benefits of different neoadjuvant chemotherapy regimens, and we can identify no phase 3 randomised trials that have directly compared different regimens.

**Added value of this study**

To our knowledge, this is the largest randomised trial investigating whether an alternative neoadjuvant chemotherapy regimen might offer a survival benefit over the standard of two cycles of cisplatin and fluorouracil (CF). This trial showed that giving four cycles of epirubicin, cisplatin, and capecitabine (ECX) neoadjuvantly might increase the level of tumour regression, but does not lead to any survival benefit.

**Implications of all available evidence**

For patients with oesophageal adenocarcinoma, two cycles of CF should remain the standard choice of neoadjuvant chemotherapy regimen. Further research is ongoing into the use of neoadjuvant chemoradiotherapy, but little evidence exists that directly compares it to neoadjuvant chemotherapy.

High-quality data on the effect of neoadjuvant chemotherapy or chemoradiotherapy on health-related quality of life (HRQL) are scarce; more information is needed for clinical decision making, where small survival benefits might only be achieved with detrimental effects on many aspects of HRQL.

The results of the previous Medical Research Council (MRC) OE02 randomised clinical trial^19^ showed a survival advantage at 2 years with neoadjuvant chemotherapy and surgery over surgery alone (43% *vs* 34%; 9% increase [95% CI 3–14]), with a hazard ratio (HR) of 0·79 (95% CI 0·67–0·93), so two-cycle cisplatin with fluorouracil was used for the control group in this study. The randomised clinical MRC MAGIC trial^25^ assessed a regimen of three cycles of preoperative chemotherapy (epirubicin, cisplatin, and fluorouracil) followed by surgery and three postoperative cycles of chemotherapy in patients with gastric and oesophageal adenocarcinoma. The results showed a 5-year survival of 36% (95% CI 30–43) for perioperative chemotherapy and surgery compared with 23% (17–29) for surgery alone (overall survival HR 0·75, 95% CI 0·60–0·93).^25^ Because 45% of participants in the MAGIC trial (34% of people who had surgery) did not receive postoperative treatment (a similar pattern was also seen in the FFCD-FNCLCC 9703 trial^22^), we concluded that further assessment of perioperative chemotherapy would be challenging and it was decided that increased neoadjuvant therapy would be the best strategy. Oral capecitabine is readily available and has been shown to be equivalent to intravenous fluorouracil in colorectal cancer,^26^ so we decided to investigate four cycles of neoadjuvant epirubicin, cisplatin, and capecitabine (ECX) as the investigational group without postoperative chemotherapy. Given the results of the MAGIC trial, increasing the duration of neoadjuvant chemotherapy from two cycles to four cycles and adding anthracycline to the established doublet regimen was considered the most promising strategy for improving outcomes in patients with oesophageal adenocarcinoma in this study. A regimen of four cycles of ECX was selected because in studies of patients with advanced disease, up to 50% of patients develop resistant disease after 24 weeks of treatment and most responses have occurred by four cycles.^27^

The aims of the OE05 trial were therefore to investigate whether four cycles of preoperative ECX improves survival and HRQL compared with standard two-cycle cisplatin and fluorouracil (CF) in patients with locally advanced resectable adenocarcinoma of the oesophagus.

## Methods

### Study design and participants

The UK MRC OE05 study is an open-label, phase 3, randomised clinical trial done in 72 hospitals across the UK. Participants were of any age with surgically resectable histologically verified adenocarcinoma of the oesophagus (including Siewert types 1 and 2 gastro-oesophageal junction tumours) stage cT1N1, cT2N1, cT3N0/N1, or cT4N0/N1 where invasion was thought to be confined to diaphragm, crura, or mediastinal pleura and surgically resectable (Union for International Cancer Control [UICC] TNM staging^28^). Additionally, patients had to meet the following criteria: WHO performance status 0 or 1 and adequate respiratory and cardiac function (forced expiratory volume in 1 sec of >1·5 L and cardiac ejection fraction of ≥50% on echocardiography or multigated acquisition scan) within 4 weeks of randomisation. Within 1 week of randomisation, liver function tests needed to be at most 1·5-times normal, white blood cell count at least 3 × 10^9^ cells per L, platelet counts at least 100 × 10^9^ platelets per L, and the calculated or measured glomerular filtration rate at least 60 mL/min.

Assessment of disease stage required a contrast-enhanced multislice CT scan from neck to pelvis and endoscopic ultrasonography within 4 weeks of randomisation. Staging laparoscopy with or without peritoneal cytology and PET scanning were optional according to local practice. The final staging of patients (and Siewert classification) was done on the basis of a multidisciplinary team discussion following endoscopy, endoscopic ultrasonography, CT, and laparoscopy if appropriate.

Patients were ineligible if investigations indicated blood-borne metastases (radiologically assessed), peritoneal dissemination, local invasion involving the tracheobronchial tree, aorta, pericardium or lung, or abdominal para-aortic lymphadenopathy greater than 1 cm in diameter on CT scan or more than 6 mm in diameter on endoscopic ultrasonography. Patients were also excluded if they had received any previous treatment for oesophageal cancer, had Siewert type 3 cancer, a medical condition that was likely to compromise the proposed trial treatment. Uncontrolled angina pectoris, myocardial infarction in the 6 months before entry into the trial, heart failure, clinically significant uncontrolled cardiac arrhythmias, or any patient with a clinically significant abnormal ECG, as well as patients with abnormal left ventricular ejection fraction (LVEF) diagnosed on MUGA scan or echocardiography, including areas of abnormal contractility, were excluded. Patients with positive serology for HIV or hepatitis C, active hepatitis B, or were pregnant were also excluded.

Participating centres were considered eligible if they had a multidisciplinary team structure, had experience of two-phase oesophagectomy with two-field lymphadenectomy (unproctored surgeons were recommended to have done a minimum of 12 such operations before joining the trial), access to multislice CT and endoscopic ultrasonography equipment, and had pathologists who were experienced in reporting oesophageal cancer.

The protocol was approved by the South West Multicentre Research Ethics Committee (04/6/005) and all patients gave written consent for participation in the study. Blood and tissue samples were collected for potential future translational studies (Trans-OE05). The protocol had five amendments during the course of the trial, predominantly for administrative reasons or to add clarity; amendments did not affect the eligibility or treatment of patients. The most recent version of the protocol (version 6.0) is available online.

### Randomisation and masking

Eligible patients were enrolled by staff at participating centres who then called the randomisation line at the MRC Clinical Trials Unit at UCL (London, UK). Patients were randomly assigned (1:1) to either two cycles of CF or four cycles of ECX. Allocation was done using a computerised minimisation program, with a random element, and stratified by centre and tumour stage. No masking to treatment allocation was done because of the difference in the number of chemotherapy cycles in the two groups.

### Procedures

The control group chemotherapy regimen was two 3-weekly cycles of cisplatin and fluorouracil. Cisplatin (80 mg/m^2^) was infused intravenously on day 1 and fluorouracil (1 g/m^2^ per day) was administered intravenously on days 1–4 (total 4 g/m^2^).

The investigational chemotherapy was four 3-weekly cycles of ECX. Epirubicin (50 mg/m^2^) and cisplatin (60 mg/m^2^) were given intravenously on day 1 and capecitabine (1250 mg/m^2^ daily) was given orally continuously over all four cycles (for a total of 12 weeks).

Protocols for chemotherapy dose modifications were provided for haematological, renal, neurological, and hepatic toxicities, and for palmar–plantar erythema, stomatitis, diarrhoea, nausea, and vomiting. Toxicities were reported according to the National Cancer Institute Common Toxicity Criteria, version 3.0.^29^

Surgery was done within 4–6 weeks of completion of chemotherapy. Surgery was a two-phase oesophagectomy with a two-field (abdomen and thorax) lymphadenectomy. Centres were allowed to use a minimal access surgery approach after providing evidence that complication rates and lymph node yields were similar to an open surgery approach from at least 20 patients who had had minimal access oesophagectomy.

The stomach was the preferred conduit for reconstruction based on the right gastric and right gastroepiploic vessels. Lymph node clearance in the abdomen was at the origin of the left gastric artery and along the hepatic and splenic arteries, the upper lesser curve, and at the right and left cardiac lymph node stations. Dissection at the diaphragm was used to minimise a positive radial or circumferential resection margin by inclusion of sufficient crural fibres and a cuff of diaphragm based on endoscopic ultrasonography and intraoperative appearances. Pyloroplasty, pyloromyotomy, the placement of a feeding jejunostomy, and drain insertions were all according to local practice.

Either a right or left thoracic approach was permitted. Lymph node dissection involved para-oesophageal, subcarinal, and bronchial lymph node stations, and thoracic duct ligation just above the diaphragm was advised. The extent of proximal lymphadenectomy for the upper paraoesophageal nodes was determined by the extent of division of the oesophagus (ie, the length of oesophagus remaining after resection).

Reconstruction was recommended above the aortic arch for the right-sided approach and just below it for the left, and preferably within 5 cm of the thoracic inlet. The anastomotic technique and method of chest drainage was according to local practice. Trans-hiatal surgery was not permitted.

Postoperative complications, described as none, present but not life threatening, or life threatening, were recorded for each patient. These complications were reviewed clinically, and assigned to categories.

Processing of the surgical resection specimens and histopathological assessment of all materials were done according to the recommendations of the Royal College of Pathologists:^30^ R0, no tumour cells remaining within 1 mm of any resection margin; R1, microscopically visible positive margin indicating the presence of tumour cells at or within 1 mm of a longitudinal or radial or circumferential resection margin; and R2, macroscopically visible tumour remaining. Resection specimens were classified using UICC TNM, 6th edition.^28^ Chemotherapy-induced changes in the primary tumour were graded according to Mandard and colleagues.^31^ The Mandard tumour regression grading (TRG) system was developed originally for the assessment of squamous cell carcinoma after chemoradiation, and was used in this study because UK pathologists are familiar with this system and it has been shown to be equivalent to the Becker grading system in terms of both interobserver agreement (ie, likelihood of two pathologists concluding the same grading by their independent assessments) and prognostic ability.^32^, ^33^

Pathology data were collected from local pathologists at each centre, and used for the primary analyses of all pathology data. A second review of the Mandard primary TRG was done to assess data quality and the differences between local and central assessments of TRG. One of three experienced pathologists (HIG, RL, and SP) independently reviewed all resection slides and provided a new Mandard TRG. If the reviewing pathologist's TRG agreed with the local pathologist assessment, this TRG was accepted as the true grade. If the reviewing pathologist disagreed, a second, and if necessary a third, pathologist reviewed the slides to reach a majority decision on the Mandard TRG. These centrally reviewed data form a secondary analysis of TRG.

All participating patients were asked to complete the validated generic cancer questionnaire (European Organisation for Research and Treatment of Cancer [EORTC] QLQ-C30)^34^ and the disease-specific oesophageal module (EORTC QLQ-OES18)^35^ at baseline, day 14 of chemotherapy cycle two, day 14 of cycle four (ECX group only), immediately before surgery, at 6 weeks, at 3, 6, 9, 12, 18, and 24 months after surgery, then annually. Patients could complete questionnaires at home and bring them to the hospital, return them by post, or complete them at their clinical follow-up visit. The HRQL protocol prespecified four domains of HRQL to be examined in the primary analyses and full details of all scales and items assessed in the questionnaires will be reported subsequently. Clinical follow-up involved the same schedule with the use of tests to establish recurrent disease at the discretion of individual centres.

### Outcomes

The primary outcome measure of the trial was overall survival. Secondary outcomes were disease-free survival, effects on the primary tumour (as assessed by Mandard TRG), HRQL, and morbidity related to chemotherapy and surgery. Exploratory outcomes were progression-free survival (where a progression-free survival event was defined as the first confirmed local or distant recurrence, or death from any cause), and an analysis of Mandard TRG using an amended responder definition.

### Statistical analysis

The sample size calculations were based on the primary outcome measure of overall survival. On the basis of the OE02 trial results,^19^ which showed an absolute survival difference of 8% at 3 years, we thought it unlikely that any greater difference would be seen in this study. The trial was originally designed to require 1300 patients and 860 events, with recruitment over 6 years and a minimum follow-up period of 6 months. With a 5% two-sided significance level, this sample would provide 90% power to detect an 8% difference (or 80% power to detect a 7% difference) in 3-year survival, from 30% in the CF group. In 2007, following a period of lower than expected recruitment, the sample size was reassessed to ensure that an adequately powered study would still be achieved in a given timeframe. The updated sample size calculations required 842 patients with 677 events over 6 years with a minimum follow-up of 2 years before analysis, to provide at least 82% power to detect an 8% difference (and 70% to detect a 7% difference) at 3 years with a two-sided significance level of 5%.

Although we anticipated that the required events would be obtained 2 years after the final patient was randomly assigned, they accrued far more slowly than expected. 3 years after the final randomisation, a conditional survival analysis was done to assess the probability that continuing to wait for the full number of events would lead to a different trial conclusion. After discussion with the independent data monitoring committee and trial steering committee, a decision was reached that the current data were sufficiently robust for full analysis and dissemination, because the chance of obtaining a different conclusion with more events was less than 5%.

All safety and primary analyses were done on an intention-to-treat basis. Overall survival was calculated from the date of group assignment to the date of death. Patients either lost to follow-up or still alive at the time of analysis were censored at the date they were last known to be alive.

Because patients can only be deemed disease free after surgery, and to account for the different durations of preoperative chemotherapy, disease-free survival was analysed using a landmark analysis.^36^ Rather than the date of randomisation, the time 1 week after the last patient had surgery was used as the start point, up to a maximum of 6 months from group assignment. Patients who had an event before this point, who did not have surgery, or who were not macroscopically disease free at surgery, were said to have had an event on day 1. If any patients received surgery more than 6 months after randomisation, they were assumed to be disease free at the 6-month start point. Disease-free survival was calculated as the time from this modified origin until the first date of confirmed local recurrence, distant metastases, or death from any cause. Patients who had no evidence of relapse were censored on the last date they were known to be disease free.

Progression-free survival was calculated from random assignment to the date of the event or, in event-free patients, the date last known to be alive and free from recurrence. Overall, disease-free, and progression-free survival were compared using the log-rank χ^2^ test. The proportion of patients who survived and median survival times were estimated from a flexible parametric survival model^37^ and presented with 95% CIs. HRs were calculated from Cox proportional hazard models, including adjustment for randomisation stratification factors. Proportional hazards assumptions were tested using the Grambsch-Therneau test. The heterogeneity of treatment effects across levels of prespecified patient characteristics (sex, age, performance status, clinical T-stage, and clinical N-stage) were explored using Cox proportional hazard models.

To analyse tumour regression, the original five-point Mandard scale was dichotomised into responders (Mandard TRG 1–3) and non-responders (Mandard TRG 4–5). A second, unplanned analysis was also done because emerging evidence suggested that a more appropriate dichotomisation was Mandard TRG 1–2 as responders and grades 3–5 as non-responders.

Formal comparisons and summaries of HRQL function and symptom scales were restricted to a number of prespecified outcome measures. Global HRQL and prespecified symptoms related to the effects of chemotherapy and surgery (appetite loss, dysphagia, pain, and reflux) were compared immediately before surgery, and 3, 12, and 24 months after surgery, using an ANOVA with adjustment for baseline score. These timepoints were considered separately, rather than using a repeated measures method of analysis.

Comparisons between chemotherapy toxicities, surgical complications, tumour regression, and resection were done using a χ^2^ test or Fisher's exact test, as appropriate. A p value of less than 0·05 was used as the significance threshold. For all analyses, no attempts were made to impute values for missing data. All analyses were done using Stata, version 14.

This trial is registered with the ISRCTN registry (registration number 01852072) and ClinicalTrials.gov (NCT00041262).

### Role of the funding source

Cancer Research UK approved the study design, but had no role in data collection, data analysis, data interpretation, or writing of the report. Roche supplied capecitabine for use in the study, and had sight of the draft trial manuscript, but had no role in any other aspect of the study. MN had access to the raw data. The corresponding author had final responsibility for the decision to submit for publication.

## Results

**Figure 1:**
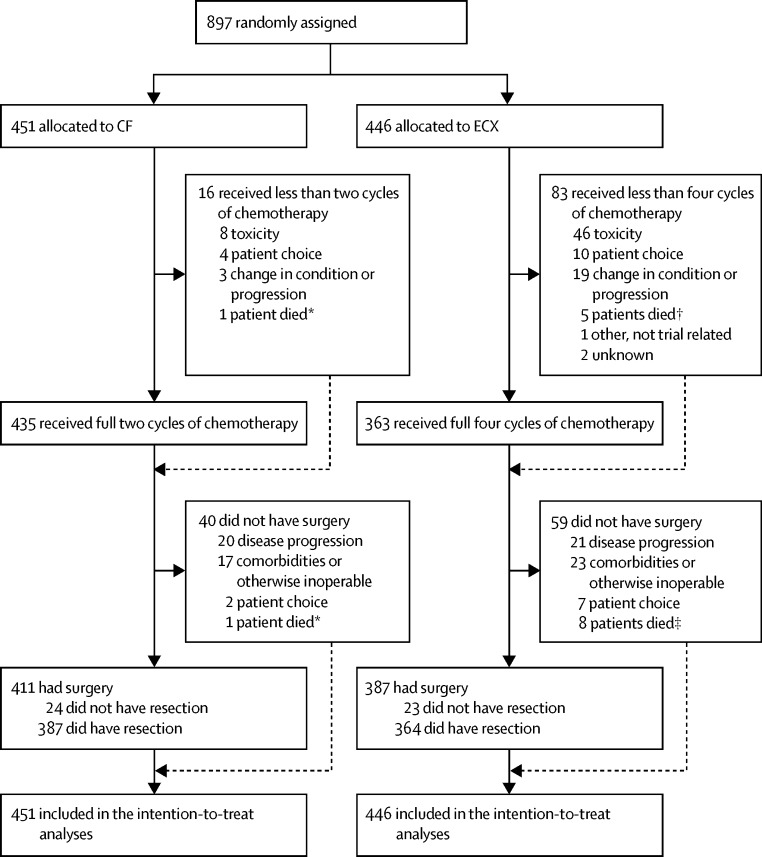
Consort diagram CF=cisplatin and fluorouracil. ECX=epirubicin, cisplatin, and capecitabine. *Cause of death reported as cardiac failure. †Causes of death reported as cerebral vascular accident, multiple organ failure, pulmonary embolism, bronchopneumonia, and oesophageal cancer. ‡For patients not listed in † above, cause of death was reported as sepsis-related multiple organ failure in one patient and oesophageal cancer in the remaining two patients. Screening logs were not collected during the trial, so the number of potentially eligible patients is unknown.

Between Jan 13, 2005, and Oct 31, 2011, 897 patients were recruited from 72 UK hospitals and randomly allocated to the CF group (n=451) or the ECX group (n=446; figure 1). The median number of patients per centre was eight (range 1–73; appendix pp 1–3). After chemotherapy, following retrospective review of the baseline CT scan, one patient was found to be ineligible because of adrenal metastases so did not have surgery, but was included in all summaries and analyses. The baseline characteristics of the patients allocated to the CF or ECX groups were similar (table 1). The median age was 62 years (IQR 56–67; range 27–81), 810 (90%) of 897 patients were male, 603 (67%) had a WHO performance status of 0, and 576 (64%) had stage T3N1 cancer (table 1; appendix pp 4–5).

**Table 1:** Baseline characteristics

	**Cisplatin and fluorouracil (n=451)**	**Epirubicin, cisplatin, and capecitabine (n=446)**
**Sex**		
Female	39 (9%)	48 (11%)
Male	412 (91%)	398 (89%)
**Age, years**		
Median (IQR)	62 (57–67)	62 (56–67)
Range	27–81	33–80
**WHO performance status**		
0	311 (69%)	292 (65%)
1	140 (31%)	154 (35%)
Stage of tumour*		
T1 N1	3 (1%)	5 (1%)
T2 N1	49 (11%)	41 (9%)
T3 N0	97 (22%)	99 (22%)
T3 N1	287 (64%)	289 (65%)
T4 N0†	3 (1%)	1 (<1%)
T4 N1†	12 (3%)	11 (2%)
**Participating in quality-of-life assessment**		
Yes	435 (96%)	424 (95%)
No	16 (4%)	22 (5%)

Data are n (%) unless otherwise specified.

* Stage of tumour (based on both endoscopic ultrasonography and CT results) was used to stratify randomisation.

† Specific site of T4 invasion is mediastinal pleura for eight patients (four in the cisplatin and fluorouracil group *vs* four in the epirubicin, cisplatin, and capecitabine group), crura for 14 patients (nine *vs* five), and diaphragm for five patients (two *vs* three).

Three (4%) of 72 recruiting centres did not take part in the HRQL aspect of the trial for any of their patients, and HRQL assessment data were omitted at baseline for the patients from these centres (37 [4%] of the total 897 patients). Baseline HRQL was also well balanced between the two groups. Mean values (SD) for the five prespecified domains of interest were: global HRQL 76·0 (18·34), appetite loss 37·4 (27·98), reflux 17·3 (24·05), pain 20·3 (20·50), and dysphagia 73·9 (25·7); data per treatment group are in the appendix (pp 8–10). A higher score indicates better HRQL for the global score and dysphagia, but worse HRQL for the symptom scales.

Details of the chemotherapy received are shown in table 2. Four patients (<1%; two in the CF group and two in the ECX group) of 897 withdrew consent before starting chemotherapy and five (1%; one CF, four ECX) died during chemotherapy. The number of patients who completed their allocated treatment was greater in the CF group than in the ECX group (435 [96%] of 451 *vs* 363 [81%] of 446; p<0·0001), although a similar number of patients in the CF (435 [96%] of 451) and ECX (432 [97%] of 446) groups received at least two cycles. Of the 451 patients in the CF group, eight (2%) stopped chemotherapy because of toxicity and one (<1%) died, whereas in the ECX group, 46 (10%) of 446 patients stopped because of toxicity, and five (1%) died, one of which was thought to be related to chemotherapy toxicity (figure 1). The number of patients requiring dose reduction to any drug was smaller in the CF than in the ECX group (88 [20%] of 451 *vs* 187 [42%] of 446; p<0·0001), although the number receiving cisplatin dose reductions was similar between the groups (61 [14%] of 451 in the CF group and 53 [12%] of 446 in the ECX group).

**Table 2:** Chemotherapy details

	**Cisplatin and fluorouracil: two cycles (n=451)**	**Epirubicin, cisplatin, and capecitabine: four cycles (n=446)**
**Cycles started**		
None*	2 (<1%)	2 (<1%)
One	14 (3%)	12 (3%)
Two (completed two-cycle regimen)	435 (96%)	32 (7%)
Three	NA	37 (8%)
Four (completed four-cycle regimen)	NA	363 (81%)
Total completed chemotherapy	435 (96%)	363 (81%)
**Delays to cycles**†		
Yes	113 (25%)	148 (33%)
No	336 (75%)	296 (66%)
No chemotherapy received	2 (<1%)	2 (<1%)
**Reduction in cisplatin dose**‡		
No	373 (83%)	378 (85%)
Yes	61 (14%)	53 (12%)
No cisplatin given	1 (<1%)	1 (<1%)
Less than two cycles given	16 (4%)	14 (3%)
**Reduction in any dose**‡		
No	347 (77%)	245 (55%)
Yes	88 (20%)	187 (42%)
Less than two cycles given	16 (4%)	14 (3%)

Data are n (%). NA=not applicable.

* All four patients who received no on-trial chemotherapy withdrew consent soon after randomisation.

† A delay is defined as a cycle starting at least 25 days after the previous cycle, or at least 11 days after randomisation.

‡ A reduction is defined as the dose decreasing by more than 10% compared with cycle one. A cycle was said to have started if any drug was administered.

The median time from randomisation to surgery was 71 days (IQR 66–80) in the CF group and 127 days (119–137) in the ECX group. The median time of surgery from the start of the last preoperative chemotherapy cycle was 44 days (39–52) for patients in the CF group and 57 days (52–64) for patients in the ECX group. Details of the surgical procedure are shown in table 3. Of the 897 patients who were enrolled, 99 (11%) did not have surgery. Of the 99 who did not have surgery, 41 (41%) were because of disease progression, nine (9%) died before surgery, nine (9%) decided not to have surgery, 15 (15%) developed significant comorbidities that precluded surgery, and 25 (25%) were deemed otherwise unsuitable for surgery. In total, 411 (91%) of 451 patients in the CF group and 387 (87%) of 446 patients in the ECX group proceeded to surgery (figure 1).

**Table 3:** Surgery details

	**Cisplatin and fluorouracil (n=451)**	**Epirubicin, cisplatin, and capecitabine (n=446)**
**Surgery done**		
Yes	411 (91%)	387 (87%)
No	40 (9%)	59 (13%)
Reason for no surgery*		
CT evidence of disease progression	13 (33%)	11 (19%)
Clinical evidence of disease progression	3 (8%)	6 (10%)
Laparoscopic evidence of disease progression	4 (10%)	4 (7%)
Comorbidity	6 (15%)	9 (15%)
Patient choice	2 (5%)	7 (12%)
Patient died	1 (3%)	8 (14%)
Patient otherwise deemed inoperable	11 (28%)	14 (24%)
**Resection done**		
Yes	387 (86%)	364 (82%)
No (open-close operation)	24 (5%)	23 (5%)
**Surgical approach**†		
Abdomen and right chest open	192 (50%)	187 (51%)
Abdomen (laparoscopic) and right chest open	108 (28%)	101 (28%)
Left thoracoabdominal incision	28 (7%)	24 (7%)
Totally laparoscopic	9 (2%)	9 (2%)
Other	43 (11%)	35 (10%)
Missing	7 (2%)	8 (2%)
**Location**†		
Mid-oesophagus	72 (19%)	56 (15%)
Siewert type 1	227 (59%)	208 (57%)
Siewert type 2	76 (20%)	89 (24%)
Missing	12 (3%)	11 (3%)

Data are n (%). An open-close operation was deemed as one in which no resection was done, or the reason given on the case report form for not having surgery was that the patient was found to be inoperable at laparotomy or thoracotomy.

* Percentages are out of patients who did not have surgery.

† Percentages are out of all patients who did have resection.

751 (84%) of 897 people had resection and reconstruction, whereas 47 (5%) of 897 patients were found to have progressive disease or to be inoperable during surgery (table 3). 588 (78%) of 751 resections were done via the abdomen and right chest using open surgery throughout (Ivor-Lewis resection) or as hybrid with a laparoscopic abdominal approach. Only 18 (2%) of the 751 resections (nine in each group) were done with a totally minimally invasive approach.

**Figure 2:**
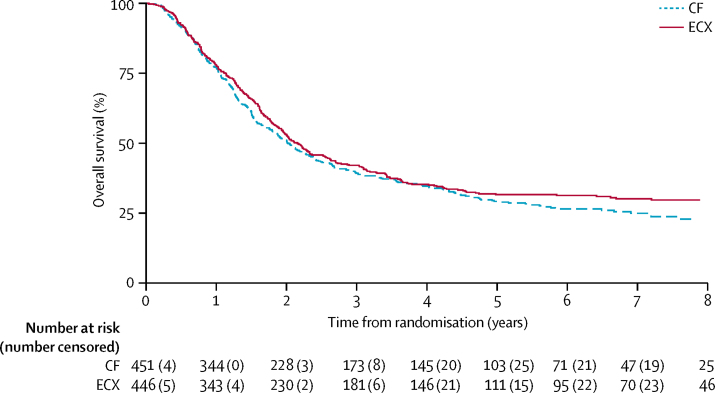
Overall survival CF=cisplatin and fluorouracil. ECX=epirubicin, cisplatin, and capecitabine.

As of Nov 14, 2016, 629 (70%) of 897 patients were known to have died (327 [73%] of 451 in the CF group and 302 [68%] of 446 in the ECX group) and a further 12 had withdrawn consent for further follow-up. The median follow-up of the surviving patients was 6·4 years (IQR 4·8–8·2), and 250 (93%) of 268 patients had at least 3 years of follow-up assessments. The observed 3-year overall survival was 39% (95% CI 35–44) in the CF group, and 42% (37–47) in the ECX group (figure 2). Median overall survival was estimated to be 23·4 months (95% CI 20·6–26·3) in the CF group and 26·1 months (22·5–29·7) in the ECX group, with an HR of 0·90 (95% CI 0·77–1·05, p=0·19). No evidence indicated that the proportional hazards assumption was violated.

**Figure 3:**
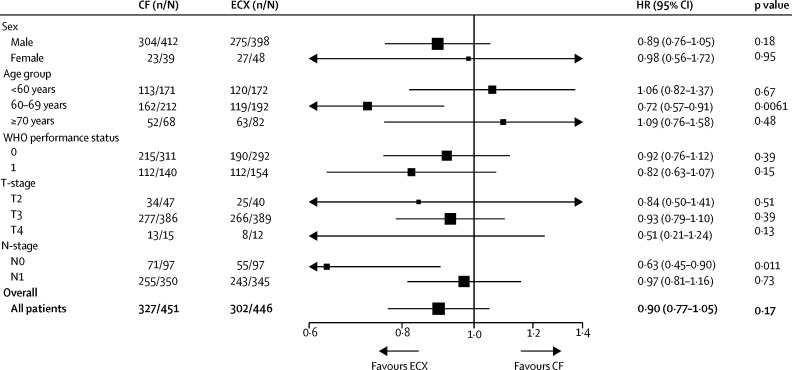
Subgroup analysis Data are number of patients who had a survival event (n) out of the total number of patients (N), or HR (95% CI). The subgroup of patients with T1 disease at randomisation is not shown because there were only three patients who had CF and five who had ECX treatment. p values for heterogeneity of treatment effect are 0·69 for sex, 0·05 for age, 0·46 for WHO performance status, 0·11 for T-stage, and 0·028 for N-stage. T-stage and N-stage refer to clinical staging collected at time of randomisation. CF=cisplatin and fluorouracil. ECX=epirubicin, cisplatin, and capecitabine.

Figure 3 shows prespecified subgroup analyses of overall survival were done, considering sex, age group (<60 years, 60–69 years, and ≥70 years), WHO performance status, clinical T-stage, and clinical N-stage (appendix p 11).

Median disease-free survival (347 events in the CF group *vs* 316 events in the ECX group, based on a 6-month landmark analysis; appendix p 13) was 11·6 months (95% CI 8·9–13·3) in the CF group and 14·4 months (11·7–16·5) in the ECX group, with an HR of 0·86 (95% CI 0·74–1·00, p=0·051).

Chemotherapy toxicity data were not provided by three patients in each group and two in each group did not receive any chemotherapy. Grade 3 or 4 diarrhoea occurred in six (1%) of 446 people in the CF group and 36 (8%) of 441 in the ECX group (p<0·0001) and grade 3 or 4 neutropenia occurred in 74 (17%) of 446 in the CF group and 101 (23%) of 441 people in the ECX group (p=0·023); stomatitis occurred in 25 (6%) of 446 people in the CF group versus seven (2%) of 441 in the ECX group (p=0·0018; table 4 and appendix p 6).

**Table 4:** Chemotherapy toxicity

	Cisplatin and fluorouracil: two cycles (n=446*)			Epirubicin, cisplatin, and capecitabine: four cycles (n=441*)		
	Grade 1–2	Grade 3	Grade 4	Grade 1–2	Grade 3	Grade 4
Neutropenia	102 (23%)	57 (13%)	17 (4%)	119 (27%)	79 (18%)	22 (5%)
Deep vein thrombosis or pulmonary embolism	5 (1%)	4 (1%)	5 (1%)	11 (2%)	8 (2%)	11 (2%)
Vomiting	116 (26%)	20 (4%)	0	177 (40%)	26 (6%)	0
Nausea	245 (55%)	16 (4%)	0	282 (64%)	27 (6%)	0
Diarrhoea	103 (23%)	6 (1%)	0	132 (30%)	33 (7%)	3 (1%)
Plantar–palmar erythrodysesthesia	27 (6%)	0	0	169 (38%)	37 (8%)	1 (<1%)
Stomatitis	212 (48%)	25 (6%)	0	199 (45%)	7 (2%)	0
Infection or febrile neutropenia	5 (1%)	2 (<1%)	1 (<1%)	6 (1%)	14 (3%)	0
Cardiac toxicity	13 (3%)	1 (<1%)	1 (<1%)	17 (4%)	2 (<1%)	1 (<1%)
Peripheral neuropathy	27 (6%)	1 (<1%)	0	103 (23%)	3 (1%)	0
Loss of taste	147 (33%)	2 (<1%)	0	180 (41%)	1 (<1%)	0
Thrombocytopenia	28 (6%)	2 (<1%)	0	32 (7%)	0	1 (<1%)
Renal toxicity	28 (6%)	2 (<1%)	0	37 (8%)	1 (<1%)	0
Tinnitus	86 (19%)	2 (<1%)	0	71 (16%)	0	0
Liver toxicity	20 (4%)	0	0	33 (7%)	2 (<1%)	0
Alopecia	84 (19%)	0	0	314 (71%)	0	0
Other toxicity	274 (61%)	36 (8%)	3 (1%)	300 (68%)	60 (14%)	7 (2%)

Data are n (%).

* Two patients in each group did not receive any chemotherapy, and another three patients in each group did not provide any toxicity data. If some toxicity data were provided, any missing toxicity data at that cycle are assumed to indicate that toxicity did not occur. One patient in the epirubicin, cisplatin, and capecitabine group died of cerebrovascular incident (reported as other toxicity).

More patients in the ECX group (108 [24%] of 446) reported serious adverse events over the course of the trial than in the CF group (73 [16% of 451], p=0·003; appendix p 7). Particularly, reports of diarrhoea were more common in the ECX group (14 [3%]) than in the CF group (0, p<0·0001). No significant differences were measured between the groups for any other type of event, or in any specific body systems (appendix p 7).

In patients for whom data were reported, no difference was seen in the overall prevalence of surgical complications between the two treatment groups (224 [56%] of 398 people in the CF group and 233 [62%] of 374 in the ECX group; p=0·089), although more people in the ECX group had respiratory complications (125 [33%] of 374) than in the CF group (107 [27%] of 398; p=0·048; appendix p 7). At 30 days after surgery, ten (2%) of 411 patients in the CF group and 11 (3%) of 387 people in the ECX group had died, and at 90 days, 21 (5%) people in the CF group and 23 (6%) people in the ECX group had died.

In the local pathologist review (table 5), the primary tumour was classified as Mandard TRG 1–3 in 44 (15%) of the 288 specimens with available results from the CF group and 93 (32%) of the 289 specimens from the ECX group (p<0·0001). Mandard primary tumour regression data were also available from central pathology review of 656 patients (87% of patients who had a resection) and a total of 24 625 slides were received, with a median of 34 (IQR 24–45) slides per person. Similar incidences of tumour regression were seen with 91 (29%) of 317 specimens graded as TRG 1, 2, or 3 in the ECX group and 40 (12%) of 339 in the CF group (p<0·0001).

**Table 5:** Pathology details

	Cisplatin and fluorouracil (n=387)*	Epirubicin, cisplatin, and capecitabine (n=364)*
**Type of tumour**		
Squamous	5/386 (1%)	1/360 (<1%)
Adenocarcinoma	370/386 (96%)	336/360 (93%)
Other	11/386 (3%)	23/360 (6%)
**Differentiation**		
Well	25/379 (7%)	28/336 (8%)
Moderate	167/379 (44%)	158/336 (47%)
Poor	187/379 (49%)	150/336 (45%)
**Mandard tumour regression grade (local pathology assessment)**		
1: Complete regression	9/288 (3%)	32/289 (11%)
2: Mainly fibrosis	9/288 (3%)	16/289 (6%)
3: Increased residual cancer cells	26/288 (7%)	45/289 (16%)
4: Residual cancer cells outgrowing fibrosis	104/288 (9%)	97/289 (34%)
5: Absence of regressive changes	140/288 (49%)	99/289 (34%)
**Mandard tumour regression grade (central pathology review)**		
1: Complete regression	5/339 (1%)	21/317 (7%)
2: Mainly fibrosis	7/339 (2%)	16/317 (5%)
3: Increased residual cancer cells	28/339 (8%)	54/317 (17%)
4: Residual cancer cells outgrowing fibrosis	194/339 (57%)	164/317 (52%)
5: Absence of regressive changes	105/339 (31%)	62/317 (20%)
**Complete resection at all margins**		
No	143/379 (38%)	108/357 (30%)
Yes	236/379 (62%)	249/357 (70%)
**Staging ypT**		
0	6/383 (2%)	19/359 (5%)
1	28/383 (7%)	49/359 (14%)
2	67/383 (17%)	58/359 (16%)
3	270/383 (70%)	223/359 (62%)
4	12/383 (3%)	10/359 (3%)
**Staging ypN**		
0	115/385 (30%)	142/361 (39%)
1	232/385 (60%)	191/361 (53%)
2	28/385 (7%)	19/361 (5%)
3	10/385 (3%)	9/361 (2%)
**Staging ypM**		
0	80/378 (21%)	90/356 (25%)
1	22/378 (6%)	13/356 (4%)
X	276/378 (73%)	253/356 (71%)
**Extent of resection**		
R0: absolute curative	212/357 (59%)	223/336 (66%)
R1: relative curative	130/357 (36%)	103/336 (31%)
R2: non-curative	15/357 (4%)	10/336 (3%)

Data are n/N (%). Denominators are total specimens in which the parameter could be assessed and results were not missing. ypT=pathological T-stage. ypN=pathological N-stage. ypM=pathological M-stage. R0=no tumour cells within 1 mm of any resection margin. R1=presence of tumour cells at or within 1 mm of a longitudinal or radial or circumferential resection margin. R2=macroscopically visible tumour left behind during surgery.

* Total number of patients for whom specimens were obtained.

More specimens from the ECX group were classified by the local pathologist as ypT0 or T1 (34 [9%] of 383 CF *vs* 68 [19%] of 359 ECX; p<0·0001) after surgery and the number of patients categorised as ypN0 was also higher after ECX (115 [30%] of 385 CF *vs* 142 [39%] of 361 ECX; p=0·0070). The proportions of patients who achieved R0, R1, and R2 were similar in both groups.

No statistically and clinically relevant differences were seen between the treatment groups in terms of HRQL (appendix pp 8–10) in any of the prespecified domains (global quality of life, pain, reflux, appetite loss, or dysphagia) or at nearly all timepoints (preoperatively and 3, 12, and 24 months postoperatively). Global HRQL was lower preoperatively during chemotherapy and postoperatively, and remained lower than at randomisation throughout the trial. Appetite loss improved during chemotherapy, worsened postoperatively, before returning to preoperative levels at 12 months after surgery. Dysphagia improved during the trial, and was slightly better at the preoperative assessment in the ECX group (appendix p 10) than in the CF group. Few changes were seen in pain and reflux scores throughout the study. Scores at 24 months were mostly within ten points of baseline values within the five prespecified domains of HRQL. One exception was dysphagia, in which a mean improvement of 10·5 points (SD 25·65) was seen in the ECX group at 24 months after surgery. The proportion of surviving patients who completed HRQL assessments declined over time, with 725 (84%) of 860 patients completing an assessment at randomisation, 339 (57%) of 595 at 3 months after surgery, 208 (45%) of 467 at 12 months, and 142 (44%) of 322 at 24 months.

When the trial and analyses were planned, we felt that Mandard TRG 1, 2, or 3 was the most suitable definition of good response to treatment. Exploratory analyses (appendix p 14) in patients who had available specimens suggested that postoperative survival of patients with tumours considered to be Mandard TRG 1 or 2 was significantly different (appendix p 14) to survival in those with tumours considered to be Mandard TRG 3, 4, or 5, so Mandard TRG 1 or 2 appeared to denote significant tumour regression. Applying this definition to the locally collected histopathological data, 18 (6%) of 288 patients in the CF group and 48 (17%) of 289 patients in the ECX group achieved significant tumour regression (p<0·0001). Similarly, using the central review data, 12 (4%) of 339 patients in the CF group and 37 (12%) of 317 patients in the ECX group achieved notable tumour regression (p<0·0001).

In the exploratory analysis of progression-free survival, median progression-free survival (343 events in the CF group *vs* 313 in the ECX group; appendix p 13) was 18·4 months (95% CI 15·2–20·5) in the CF group and 21·4 months (19·4–24·0) in the ECX group, with an HR of 0·84 (95% CI 0·72–0·98, p=0·033). The contributing progression-free survival event was either local recurrence (60 [17%] of 343 patients in the CF group *vs* 46 [15%] of 313 in the ECX group), distant metastases (94 [27%] of 343 people *vs* 78 [25%] of 313), local recurrence and distant metastases (87 [25%] of 343 *vs* 59 [19%] of 313), or death without confirmed progression (102 [30%] of 343 *vs* 130 [42%] of 313).

An exploratory analysis highlighted that the overall survival HR did not remain constant over time, and a strong interaction with year of randomisation was seen (p=0·0004; appendix p 12). Patients randomly assigned early in the trial (2005–07) had some survival benefit in the ECX group compared with those in the CF group, whereas those assigned in later years (2008 onwards) did not. The use of PET scans increased greatly over the course of the trial, and so a further exploratory subgroup analysis was done looking at the effect of receiving a PET scan. This subgroup analysis also showed a strong interaction with treatment group (p value=0·0008). Patients who did not have a PET scan had longer overall survival in the ECX group, whereas for patients who did receive a PET scan, ECX gave no survival advantage.

## Discussion

This study showed that more intensive neoadjuvant chemotherapy with four cycles of ECX provided no overall or disease-free survival advantage over two cycles of CF in 897 patients with oesophageal adenocarcinoma. Chemotherapy toxicity and serious adverse events were reported more often with ECX—as can be expected from four cycles of a triplet regimen compared with two cycles of a doublet regimen. These adverse events contributed to a greater number of patients completing their planned chemotherapy and undergoing surgery in the CF group than in the ECX group, although surgical resection, postoperative complications, and postoperative mortality were similar between the groups. In a post-hoc exploratory analysis, improved progression-free survival was shown with ECX treatment compared with CF treatment.

More patients in the ECX group had a good pathological response to chemotherapy (Mandard TRG 1 or 2), and consequently more were staged as ypT0 or 1 or ypN0 after surgery, than were those in the CF group. Results from the MRC gastro-oesophageal ST03^38^ study showed that Mandard TRG 1 or 2 after chemotherapy was associated with improved survival compared with Mandard TRG 3, 4, or 5. Similar exploratory analyses based on response to chemotherapy within this study also suggest a postoperative survival benefit for those patients with Mandard TRG 1 or 2. However, the absolute numbers of specimens assessed that were classified as Mandard TRG 1 or 2 (48 [17%] of 289 in the ECX group *vs* 18 [6%] of 288 in the CF group) were low, and so this difference did not translate into an overall survival benefit.

The results of this trial raise the question of the optimal number of preoperative chemotherapy cycles. In the absence of a good pathological response of the tumour, giving more than two cycles might unnecessarily delay surgery. In the current absence of a reliable biomarker that enables prediction of responses either before or midway through preoperative treatment, the extent of preoperative chemotherapy relies on the judgement of the clinician after discussion with the patient, to balance the possibility of achieving a good response against the risk of not being able to proceed to surgery. The results from our OE05 trial favour the use of two cycles of a chemotherapy doublet if preoperative chemotherapy alone is offered. However, two cycles of CF preoperatively has not been directly compared with the perioperative approach assessed in the MAGIC trial,^25^ which studied three cycles of ECX given preoperatively and postoperatively. The FFCD-FNCLCC 9703 trial^22^ also showed the superiority of perioperative chemotherapy over surgery alone, using the CF regimen in a population predominantly of patients with adenocarcinoma. Given the increased proportion of patients who achieved a response with ECX treatment in our study, a smaller number of preoperative cycles (two or three) with a triplet regimen combined with the selective use of postoperative chemotherapy for responding patients might be a better approach than either four cycles of ECX or two cycles of CF.

OE05 showed an improvement in overall survival of patients in the CF group (recruited 2005–11) compared with the same regimen in OE02 (recruited 1992–98), in which the median survival was 1·4 years compared with 2·0 years in OE05.

A number of potential factors could explain why the survival of patients with oesophageal cancer who were treated with CF improved with time. Changes in referral patterns, moves towards centralisation of surgical services, the introduction of endoscopic ultrasonography, and the development of multidisciplinary teams are all events that occurred towards the end of recruitment to the OE02 study, and these changes might have led to better patient selection for attempted curative treatment by the time of this OE05 study. The introduction of routine PET scanning might also be an important factor for the absence of survival difference in this study. Post-hoc analyses indicated that ECX offered a survival benefit in the early years of the study when PET scans were rarely used, but offered no benefit during the later years when PET was used for nearly all patients. ECX might be more effective in treating small-volume, disseminated disease that was often missed before the routine use of PET scans. Patients with this type of disease are now usually identified and would have been ineligible for participation in the study.

Neoadjuvant chemoradiotherapy has also led to improved survival compared with surgery alone for patients with oesophageal adenocarcinoma and squamous cell cancers, with a median survival of 48·6 months (95% CI 32·1–65·1) with adjuvant therapy compared with 24·0 months (95% CI 14·2–33·7) with surgery alone in the CROSS trial.^39^ The HRs for comparison with surgery alone are similar with chemoradiotherapy in CROSS (0·73 [95% CI 0·55–0·88] in patients with adenocarcinoma) and with chemotherapy in MAGIC (0·75 [0·60–0·93]), suggesting that although the trials studied slightly different populations, the size of benefit might be similar. Trials comparing these two approaches directly^20^, ^23^, ^24^ have shown an increased response with chemoradiotherapy, but without any subsequent improvement in overall survival.

The present study has a number of advantages over other oesophageal cancer trials. This study was confined to patients with adenocarcinomas of the oesophagus and gastro-oesophageal junction (Siewert type 1 and 2), specifically excluding squamous cell cancers and Siewert type 3 (gastric cancer). The study involved a large number of centres with a detailed protocol regarding pretreatment staging, the delivery of chemotherapy and surgery, and histopathological assessment. To our knowledge, our study is the only prospective randomised trial in neoadjuvant treatment and surgery for oesophageal cancer to have included a comprehensive prospective assessment of HRQL using validated generic and specific measures. Although no differences between trial groups were observed in HRQL, the data confirm findings from much smaller cohort studies.^40^ Neoadjuvant chemotherapy and surgery are associated with reduced HRQL that persists for more than 6 months after surgery, and some features such as appetite loss persist for at least 12 months. These HRQL results should be accepted as the standard that can be achieved with current platinum-based or fluoropyrimidine-based neoadjuvant chemotherapy and surgery, and communicated to patients during shared decision making before surgery, along with the likely median survival data of the combined interventions. Limitations of this study include the changing use of PET scanning through the course of this trial as we have discussed and its potential effect on the prognosis of patients who entered the trial over time. Additional limitations are the fact that postoperative chemotherapy was not assessed, and the challenge of interpreting and implementing these results in the face of the evolving role of chemoradiation in the management of oesophagogastric adenocarcinomas.

Data published in 2017 show a survival advantage for patients treated with docetaxel, oxaliplatin, and fluorouracil compared with epirubicin, cisplatin, and fluorouracil or ECX given perioperatively for junctional and gastric tumours, with a 3-year overall survival of 57% for the docetaxel-containing regimen compared with 48% with epirubicin, cisplatin, and fluorouracil or ECX (HR 0·77, 95% CI 0·63–0·94).^41^ Though an improvement in overall survival for biomarker-unselected patients, these results highlight that further improvements in outcomes are still needed for these patients. Alternative neoadjuvant approaches such as chemoradiation are also being investigated.^42^, ^43^ Additional correlative science projects are planned for this trial with the aim of identifying subsets of patients who might specifically benefit from ECX neoadjuvant chemotherapy.

For the **OE05 Clinical Trial Protocol Version 6.0** see http://www.ctu.mrc.ac.uk/research/documents/cancer_protocols/OE05_Protocol_Version_6_23_Dec_09.pdf
