# Supplementary material for: Neoadjuvant cisplatin and fluorouracil versus epirubicin, cisplatin, and capecitabine followed by resection in patients with oesophageal adenocarcinoma (UK MRC OE05): an open-label, randomised phase 3 trial
Source: Lancet Oncol. 2017 Sep;18(9):1249–60. doi: 10.1016/S1470-2045(17)30447-3 (PMC5585417; doi:10.1016/S1470-2045(17)30447-3)
Supplement: Supplementary appendix [file mmc1.pdf]

THE LANCET
Oncology

Supplementary appendix

This appendix formed part of the original submission and has been peer reviewed. We post it as supplied by the authors.

Supplement to: Alderson D, Cunningham D, Nankivell M, et al. Neoadjuvant cisplatin and fluorouracil versus epirubicin, cisplatin, and capecitabine followed by resection in patients with oesophageal adenocarcinoma (UK MRC OE05): an open-label, randomised phase 3 trial. *Lancet Oncol* 2017; published online August 4. http://dx.doi.org/10.1016/S1470-2045(17)30447-3.

A randomised trial comparing neoadjuvant cisplatin/5-FU with epirubicin/cisplatin/capecitabine followed by resection for oesophageal adenocarcinoma. Results from the UK Medical Research Council OE05 randomised clinical trial

Supplementary material

Acknowledgements

Trial Management Group:

Derek Alderson; Jane Blazeby; Adrian Crellin; David Cunningham; Heike Grabsch; Mike Griffin.

CTU MRC at UCL staff:

Elizabeth Armstrong; Julie Bakobaki; Claire Chandler; Christina Chung; Paul Cortissos; Carlos Diaz; Alexandra Enston; Laura Farrelly; Cindy Goldstein; Lynda Harper; Brooke Jackson; Sarah Kirk; Ruth Langley; Linda Ly; Leah Meaden; Matthew Nankivell; Laura Nichols; Dipa Noor; Sabrina Patel; Helen Pearce; Jennifer Pickard; Claire Robb; Sally Stenning; Monique Tomiczek; Barbara Uscinska; Monica Verma; Simon Weeden.

Independent Data Monitoring Committee:

John Bancewitz; Pippa Corrie; Janet Dunn; Mike Hallissey.

Trial Steering Committee:

Peter Johnson; Philip Johnson; Malcolm Mason; Chris Parker; Robin Rudd; Jeremy Whelan.

Other advisors/collaborators:

Rupert Langer; Alicia Okines; Susan Pritchard; Elizabeth Smyth;

Additional funding:

David Cunningham receives funding from The National Institute of Health Biomedical Centre at the Institute of Research and Royal Marsden Hospital.

Jane Blazeby receives funding from the Medical Research Council ConDuCT-II Hub for Trials Methodology Research at the University of Bristol.

Randomising centres

Centre	Principal Investigator			#Patients
Bristol Haematology & Oncology Centre	Dr	S	Falk	74
St James's University Hospital	Dr	AM	Crellin	50
Freeman Hospital + Newcastle General Hospital	Dr	FY	Coxon	45
Heartlands Hospital	Dr	J	Thompson	43
Royal Marsden Hospital (Sutton + London)	Prof	D	Cunningham	37
Yeovil District Hospital	Dr	E	Beaumont	34
Royal United Hospital	Mr	R	Krysztopik	30
Guy's Hospital	Dr	P	Ross	28
Queen Elizabeth Hospital	Dr	D	Peake	28
Huddersfield Royal Infirmary	Dr	J	Dent	27
Maidstone Hospital	Dr	J	Waters	23
Western General Hospital	Dr	H	Phillips	23
Royal Surrey County Hospital	Dr	S	Cummins	21
Leicester Royal Infirmary	Dr	A	Thomas	19
Peterborough City Hospital	Dr	K	McAdam	19
Queen Alexandra Hospital	Dr	C	Archer	18
Addenbrooke's Hospital	Dr	H	Ford	17
Cheltenham General Hospital	Dr	S	Elyan	17
Queens Hospital	Mr	D	Khoo	17
St Bartholomew's Hospital	Dr	S	Slater	16
University Hospital Coventry	Dr	S	Sothi	16
Cumberland Infirmary	Dr	J	Nicoll	15
Dorset County Hospital	Dr	M	Bayne	15
Princess Alexandra Hospital	Dr	J	Bridgewater	15
Weston Park Hospital	Dr	J	Wadsley	15
Royal Cornwall Hospital (Treliske)	Dr	R	Ellis	14
Royal Bournemouth General Hospital	Dr	T	Geldart	13
Velindre Hospital	Dr	T	Crosby	13
Churchill Hospital	Dr	N	Warner	12
Royal Blackburn Hospital	Dr	W	Appel	11
University Hospital of North Staffordshire	Dr	A	Jamil	11
Bradford Royal Infirmary	Dr	S	Cheeseman	9
Musgrove Park Hospital	Dr	E	Cattell	9
Aintree Hospitals Trust	Dr	H	Neville-Webb	8
Clatterbridge Centre for Oncology	Dr	A	Moss	8
Torbay District General Hospital	Dr	N	Dorey	8
University College London	Dr	D	Hochhauser	8
Derbyshire Royal Infirmary	Dr	RB	Kulkarni	7
Derriford Hospital	Dr	S	Pascoe	7
Great Western Hospital	Dr	C	Blessing	6
Kent and Canterbury Hospital	Dr	J	Waters	6

Centre	Principal Investigator			#Patients
Worcestershire Royal Hospital	Dr	C	Candish	6
Glan Clwyd Hospital	Dr	A	Garcia-Alonso	5
Good Hope Hospital	Dr	J	Glaholm	5
Royal Devon & Exeter (Wonford) Hospital	Dr	E	Toy	5
The Alexandra Hospital	Dr	S	Sothi	5
Broomfield Hospital	Dr	S	Tahir	4
St Mary's Hospital (London)	Dr	D	Power	4
Weston General Hospital	Dr	S	Hilman	4
York District Hospital	Dr	K	Last	4
Burnley General Hospital	Dr	W	Appel	3
Countess of Chester Hospital	Mr	D	Monk	3
Hereford County Hospital	Dr	N	Reed	3
North Middlesex Hospital	Dr	J	Bridgewater	3
Poole General Hospital	Dr	V	Laurence	3
Royal Derby Hospital	Dr	RB	Kulkarni	3
Royal Free Hospital	Dr	T	Meyer	3
Royal Lancaster Infirmary	Dr	D	Fyfe	3
Royal Preston Hospital	Dr	E	Young	3
St George's Hospital	Dr	T	Benepal	3
Victoria Hospital	Dr	S	Susnerwala	3
Furness General Hospital	Dr	D	Fyfe	2
Whiston Hospital	Dr	E	Marshall	2
Ysbyty Gwynedd	Prof.	N	Stuart	2
Darent Valley Hospital	Dr	R	Shah	1
James Cook University Hospital	Dr	N	Wadd	1
King George Hospital	Mr	D	Khoo	1
William Harvey Hospital	Dr	J	Waters	1

Supplementary Table S1 CT staging information

Data		CF N=450		ECX N=446		Total N=896	
		N	%	N	%	N	%
**CT length of tumour (cm)**	**Median (IQR)**	5	(3·4-6)	5	(3·5-6)	5	(3·5-6)
	**Min – Max**	0–14·7		0-16		0-16	
	Missing	*119*	*26%*	*125*	*28%*	*244*	*27%*
**Extent of disease**	**T1-T3**	416	92%	414	93%	830	93%
	**T4**	11	2%	8	2%	19	2%
	Missing	*23*	*5%*	*24*	*5%*	*47*	*5%*
**CT designated malignant nodes**	**None**	223	50%	225	50%	448	50%
	**Mediastinal**	87	19%	77	17%	164	18%
	**Abdominal**	82	18%	78	17%	160	18%
	**Mediastinal & Abdominal**	49	11%	50	11%	99	11%
	Missing	*9*	*2%*	*16*	*4%*	*25*	*3%*

Note: One CF patient withdrew consent before providing any on-study staging information, and is not included in the above table.

Supplementary Table S2 EUS staging information

Data		CF N=450		ECX N=446		Total N=896	
		N	%	N	%	N	%
**EUS overall length of tumour (cm)**	**Median (IQR)**	5	(4-7)	5	(4-7)	5	(4-7)
	**Min – Max**	0·5-15		0·5-16		0·5-16	
	Missing	*52*	*12%*	*49*	*11%*	*101*	*12%*
**EUS oesophageal involvement (cm)**	**Median (IQR)**	5	(3-6)	5	(3-6)	5	(3-6)
	**Min – Max**	0-15		0·4-16		0-16	
	Missing	*90*	*21%*	*97*	*22%*	*187*	*22%*
**EUS Gastric involvement (cm)**	**Median (IQR)**	0	(0-0)	0	(0-0)	0	(0-0)
	**Min – Max**	0-6		0-6		0-6	
	Missing	*97*	*21%*	*92*	*21%*	*189*	*21%*
**Extent of disease**	**T1**	3	1%	5	1%	8	1%
	**T2**	50	11%	41	9%	91	10%
	**T3**	374	83%	382	86%	756	84%
	**T4**	13	3%	12	3%	25	3%
	Missing	10	2%	6	1%	16	2%
**EUS designated malignant nodes**	**None**	152	34%	158	35%	310	35%
	**Mediastinal**	92	20%	97	22%	189	21%
	**Abdominal**	116	26%	114	26%	230	26%
	**Med & Abd**	63	14%	55	12%	118	13%
	**Missing**	27	6%	22	5%	49	5%

Note: One CF patient withdrew consent before providing any on-study staging information, and is not included in the above table.

Supplementary Table S3 Other staging procedures (all patients received CT and EUS)

Data	CF N=450		ECX N=446		Total N=896	
	N	%	N	%	N	%
**Laparoscopy performed**	216	48%	213	48%	429	48%
**PET scan performed**	271	60%	270	61%	541	60%
**Bone scan performed**	23	5%	25	6%	48	5%
**Other staging investigations performed**	37	8%	33	7%	70	8%

Note: One CF patient withdrew consent before providing any on-study staging information, and is not included in the above table.

Supplementary Table S4 Grade 3/4/5 Chemotherapy toxicity

Data	CF – 2 cycles N=446		ECX – 4 cycles N=441		Total N=887		P-value for difference
	N	%	N	%	N	%	
**Any grade 3/4/5 toxicity**	140	31%	218	49%	358	40%	<0.0001
**Neutropenia**	74	17%	101	23%	175	20%	0.023
**DVT/PE**	19	4%	30	7%	49	6%	0.11
**Vomiting**	20	4%	26	6%	46	5%	0.37
**Nausea**	16	4%	27	6%	43	5%	0.087
**Diarrhoea**	6	1%	36	8%	42	5%	<0.0001
**Plantar-palmar erythrodysesthesia**	0	0%	38	9%	38	4%	<0.0001
**Stomatitis**	25	6%	7	2%	32	4%	0.0018
**Infection/febrile neutropenia**	3	1%	14	3%	17	2%	0.0067
**Cardiac toxicity**	4	1%	3	1%	7	1%	1.00
**Peripheral neuropathy**	1	<1%	3	1%	4	<1%	0.62
**Loss of taste**	2	<1%	1	<1%	3	<1%	1.00
**Thrombocytopenia**	2	<1%	1	<1%	3	<1%	1.00
**Renal toxicity**	2	<1%	1	<1%	3	<1%	1.00
**Tinnitus**	2	<1%	0	0%	2	<1%	0.50
**Liver toxicity**	0	0%	2	<1%	2	<1%	0.25
**Other toxicity**	39	9%	68	15%	107	12%	0.0027
**Cycle of first G3/4/5 toxicity**							
**1**	108	24%	77	17%			
**2**	32	7%	72	16%			
**3**			47	11%			
**4**			21	5%			
**None**	306	69%	224	51%			

Note: Two patients in each arm did not receive any chemotherapy. A further three patients in each arm did not provide any toxicity data. If some toxicity data were provided, any missing toxicity data at that cycle are assumed to indicate that toxicity did not take place.

Supplementary Table S5 Post-operative complications

Data	CF N=411		ECX N=387		Total N=798	
	N	%	N	%	N	%
**Any complication**	224	56%	233	62%	457	59%
**Respiratory**	107	27%	125	33%	232	30%
**Infection**	57	14%	56	15%	113	15%
**Cardiac**	44	11%	44	12%	88	11%
**Anastomotic**	45	11%	38	10%	83	11%
**Surgery related**	36	9%	42	11%	78	10%
**Haematological**	18	5%	16	4%	34	4%
**Thrombo-embolic**	16	4%	17	5%	33	4%
**Chylothorax**	12	3%	15	4%	27	4%
**Other**	27	7%	27	7%	54	7%
**Revisional operation**	34	8%	32	8%	66	8%
**Died within 90 days (based on dates)**	21	5%	23	6%	44	6%
**Died within 30 days (based on dates)**	10	2%	11	3%	21	3%

Note: Thirteen patients in each arm provided no post-operative complication data. A further ten CF and nine ECX patients did not provide follow-up data on whether a revisional operation was required, despite reporting post-operative complications.

Supplementary Table S6 Serious Adverse Events

Data	CF N=451		ECX N=446		Total N=897	
	N	%	N	%	N	%
**Any serious adverse event**	73	16%	108	24%	181	20%
**Common body-systems**						
**Gastro-intestinal**	23	5%	32	8%	55	6%
**Infection**	15	3%	18	4%	33	4%
**Cardiac**	12	3%	8	2%	20	2%
**Common events**						
**Diarrhoea**	0	0%	14	3%	14	2%
**Dysphagia**	3	1%	10	2%	13	1%
**Vomiting**	11	2%	6	1%	17	2%
**Pulmonary Embolism**	10	2%	16	4%	26	3%

Note: Only body-systems or serious adverse events reported by at least 10 patients in either arm are reported.

Supplementary Table S7 Quality of life

Analysis point	Time point	CF<br>Mean (sd), N	ECX<br>Mean (sd), N	All patients<br>Mean (sd), N	P-value
**Global Quality of Life**					
**Baseline**	**Baseline**	76·3 (19·37), 358	75·7 (17·30), 367	76·0 (18·34), 725	
**Pre-surgery**	**Baseline**<br>**Pre-surgery**<br>**Change from baseline**	76·5 (19·50), 300<br>66·5 (21·76), 300<br>-10·0 (21·24), 300	75·8 (17·66), 274<br>62·8 (24·79), 274<br>-13·0 (24·20), 274	76·1 (18·63), 574<br>64·7 (23·31), 574<br>-11·4 (22·73), 574	0·0582
**3 months post-op**	**Baseline**<br>**3 months post-op**<br>**Change from baseline**	77·0 (19·60), 177<br>60·1 (20·96), 177<br>-16·9 (23·35), 177	76·9 (16·57), 162<br>61·7 (21·65), 162<br>-15·2 (22·79), 162	76·9 (18·19), 339<br>60·9 (21·28), 339<br>-16·1 (23·06), 339	0·4566
**12 months post-op**	**Baseline**<br>**12 months post-op**<br>**Change from baseline**	80·5 (17·80), 107<br>70·0 (21·50), 107<br>-10·5 (22·29), 107	77·7 (15·77), 101<br>67·3 (20·64), 101<br>-10·4 (21·03), 101	79·2 (16·89), 208<br>68·7 (21·08), 208<br>-10·5 (21·64), 208	0·6069
**24 months post-op**	**Baseline**<br>**24 months post-op**<br>**Change from baseline**	80·1 (17·22), 67<br>73·8 (21·18), 67<br>-6·3 (24·83), 67	79·1 (15·64), 75<br>68·3 (20·64), 75<br>-10·8 (19·70), 75	79·6 (16·36), 142<br>70·9 (21·00), 142<br>-8·7 (22·30), 142	0·1358
**Appetite Loss**					
**Baseline**	**Baseline**	38·2 (28·37), 357	36·6 (27·62), 367	37.4 (27.98), 724	
**Pre-surgery**	**Baseline**<br>**Pre-surgery**<br>**Change from baseline**	38·3 (28·20), 299<br>27·6 (26·37), 299<br>-10·6 (28·64), 299	35·9 (27·88), 274<br>24·4 (26·07), 274<br>-11·5 (29·42), 274	37·1 (28·05), 573<br>26·1 (26·25), 573<br>-11·1 (28·99), 573	0·2421
**3 months post-op**	**Baseline**<br>**3 months post-op**<br>**Change from baseline**	41·0 (29·20), 175<br>36·1 (24·78), 175<br>-4·9 (34·64), 175	32·9 (25·84), 161<br>35·7 (24·24), 161<br>2·8 (29·65), 161	37·1 (27·90), 336<br>35·9 (24·49), 336<br>-1·2 (32·53), 336	0·6320
**12 months post-op**	**Baseline**<br>**12 months post-op**<br>**Change from baseline**	33·0 (27·88), 109<br>26·3 (21·67), 109<br>-6·6 (30·87), 109	33·4 (26·42), 101<br>25·7 (20·72), 101<br>-7·7 (29·78), 101	33·2 (27·12), 210<br>26·0 (21·17), 210<br>-7·1 (30·28), 210	0·8226
**24 months post-op**	**Baseline**	35·7 (30·25), 69	30·6 (26·19), 75	33·0 (28·23), 144	

Analysis point	Time point	CF<br>Mean (sd), N	ECX<br>Mean (sd), N	All patients<br>Mean (sd), N	P-value
	**24 months post-op**<br>**Change from baseline**	22·1 (22·77), 69<br>-13·65 (34·27), 69	22·9 (23·41), 75<br>-7·7 (30·50), 75	22·5 (23·03), 144<br>-10·5 (32·39), 144	0·6530
**Reflux**					
**Baseline**	**Baseline**	16·0 (22·15), 359	18·7 (25·74), 367	17·3 (24·05), 726	
**Pre-surgery**	**Baseline**<br>**Pre-surgery**<br>**Change from baseline**	16·6 (22·67), 302<br>14·1 (21·08), 302<br>-2·5 (24·47), 302	19·2 (26·01), 274<br>15·1 (22·07), 274<br>-4·0 (27·07), 274	17·8 (24·33), 576<br>14·6 (21·55), 576<br>-3·2 (25·73), 576	0·8930
**3 months post-op**	**Baseline**<br>**3 months post-op**<br>**Change from baseline**	16·0 (21·93), 177<br>24·0 (27·86), 177<br>8·0 (31·08), 177	16·5 (22·66), 159<br>21·1 (24·70), 159<br>4·6 (29·46), 159	16·2 (22·24), 336<br>22·6 (26·41), 336<br>6·4 (30·33), 336	0·2761
**12 months post-op**	**Baseline**<br>**12 months post-op**<br>**Change from baseline**	16·4 (21·51), 109<br>25·5 (26·89), 109<br>9·2 (27·92), 109	14·9 (22·35), 101<br>27·4 (29·21), 101<br>12·5 (33·20), 101	15·6 (21·88), 210<br>26·4 (27·98), 210<br>10·8 (30·54), 210	0·5254
**24 months post-op**	**Baseline**<br>**24 months post-op**<br>**Change from baseline**	13·8 (23·21), 69<br>23·4 (25·46), 69<br>9·7 (33·3), 69	17·3 (25·74), 76<br>29·8 (27·26), 76<br>12·5 (31·87), 76	15·6 (24·55), 145<br>26·8 (26·52), 145<br>11·1 (32·46), 145	0·1930
**Pain**					
**Baseline**	**Baseline**	19·3 (19·15), 359	21·3 (21·72), 367	20·3 (20·50), 726	
**Pre-surgery**	**Baseline**<br>**Pre-surgery**<br>**Change from baseline**	19·8 (19·25), 302<br>13·1 (16·72), 302<br>-6·7 (20·24), 302	21·4 (21·37), 274<br>12·0 (17·43), 274<br>-9·4 (22·23), 274	20·5 (20·28), 576<br>12·6 (17·06), 576<br>-8·0 (21·23), 576	0·2369
**3 months post-op**	**Baseline**<br>**3 months post-op**<br>**Change from baseline**	18·5 (18·72), 177<br>19·7 (19·59), 177<br>1·2 (23·35), 177	18·4 (20·30), 161<br>17·56 (17·19), 161<br>-0·86 (19·57), 161	18·5 (19·46), 338<br>18·7 (18·49), 338<br>0·2 (21·62), 338	0·2628
**12 months post-op**	**Baseline**<br>**12 months post-op**	16·1 (16·94), 109<br>15·2 (18·25), 109	18·6 (19·82), 101<br>16·6 (21·17), 101	17·3 (18·38), 210<br>15·8 (19·67), 210	0·8985

Analysis point	Time point	CF<br>Mean (sd), N	ECX<br>Mean (sd), N	All patients<br>Mean (sd), N	P-value
	**Change from baseline**	-0·9 (21·81), 109	-2·0 (20·12), 101	-1·5 (20·97), 210	
**24 months post-op**	**Baseline**<br>**24 months post-op**<br>**Change from baseline**	15·6 (19·09), 69<br>14·0 (17·64), 69<br>-1·6 (19·46), 69	17·8 (20·05), 76<br>14·3 (22·06), 76<br>-3·5 (22·16), 76	16·8 (19·56), 145<br>14·2 (20·01), 145<br>-2·6 (20·87), 145	0·8187
**Dysphagia**					
**Baseline**	**Baseline**	73·7 (25·83), 359	74·1 (25·55), 367	73·9 (25·67), 726	
**Pre-surgery**	**Baseline**<br>**Pre-surgery**<br>**Change from baseline**	73·7 (25·75), 301<br>79·4 (23·73), 301<br>5·7 (28·40), 301	75·1 (25·01), 274<br>84·4 (24·25), 274<br>9·2 (28·00), 274	74·4 (25·39), 575<br>81·8 (24·08), 575<br>7·4 (28·24), 575	0·0173
**3 months post-op**	**Baseline**<br>**3 months post-op**<br>**Change from baseline**	73·0 (26·47), 177<br>81·6 (23·06), 177<br>8·6 (32·25), 177	79·4 (22·68), 161<br>82·1 (23·27), 161<br>2·6 (29·22), 161	76·1 (24·91), 338<br>81·8 (23·12), 338<br>5·8 (30·94), 338	0·8142
**12 months post-op**	**Baseline**<br>**12 months post-op**<br>**Change from baseline**	79·3 (25·19), 109<br>88·4 (17·70), 109<br>9·1 (27·48), 109	77·6 (22·78), 100<br>85·3 (24·76), 100<br>7·7 (32·54), 100	78·5 (24·02), 209<br>86·9 (21·38), 209<br>8·5 (29·94), 209	0·3176
**24 months post-op**	**Baseline**<br>**24 months post-op**<br>**Change from baseline**	79·4 (25·16), 69<br>87·1 (23·77), 69<br>7·7 (33·46), 69	78·8 (23·53), 76<br>89·3 (19·41), 76<br>10·5 (25·65), 76	79·1 (24·23), 145<br>88·3 (21·55), 145<br>9·2 (29·55), 145	0·5183

Note: Quality of life data are collected using the EORTC QLQ-C30 and OES-18 questionnaires.
Note: Treatment groups are compared using analysis of variance, with adjustment for baseline values.

Supplementary Table S8 Survival times from planned sub-group analyses.

		CF (N=451)		ECX (N=446)	
		**N**	**Med OS (95% CI)**	**N**	**Med OS (95% CI)**
**Sex**	**Male**	412	2·00 (1·70, 2·28)	398	2·11 (1·93, 2·36)
	**Female**	39	4·03 (1·50, Undef)	48	2·63 (1·55, Undef)
**Age**	**<60**	171	2·42 (1·71, 3·67)	172	2·17 (1·85, 2·64)
	**60-69**	212	1·80 (1·52, 2·11)	192	2·33 (1·93, 3·27)
	**70+**	68	2·07 (1·70, 2·68)	82	1·80 (1·59, 2·25)
**WHO PS**	**0**	311	2·28 (2·00, 2·94)	292	2·28 (1·98, 3·03)
	**1**	140	1·50 (1·23, 1·90)	154	1·80 (1·59, 2·33)
**T-stage**	**T2**	47	2·00 (1·50, 4·48)	40	2·36 (1·64, 4·56)
	**T3**	386	2·11 (1·75, 2·42)	389	2·10 (1·85, 2·33)
	**T4**	15	1·65 (0·58, 2·06)	12	3·14 (0·80, Undef)
**N-stage**	**N0**	97	1·75 (1·28, 2·66)	97	2·89 (2·22, Undef)
	**N1**	350	2·05 (1·80, 2·40)	345	2·02 (1·82, 2·31)

Note: WHO PS = World Health Organisation Performance Status.
Note: T-stage and N-stage are as reported at time of randomisation.
Note: Undef = upper or lower limit of confidence interval is not defined.
Note: P-values for heterogeneity of treatment effect are 0·69 for sex; 0·05 for age; 0·46 for WHO PS; 0·11 for T-stage; and 0·05 for N-stage.

Supplementary Table S9 Survival times from unplanned sub-group analyses.

		CF (N=451)		ECX (N=446)	
		**N**	**Med OS (95% CI)**	**N**	**Med OS (95% CI)**
**Year of randomisation**	**2005**	42	1·67 (1·30, 2·42)	44	2·78 (1·34, 4·54)
	**2006**	74	1·44 (1·03, 2·09)	73	2·23 (1·63, Undef)
	**2007**	66	1·59 (1·33, 2·02)	63	2·64 (1·74, 4·28)
	**2008**	81	2·51 (1·51, 4·55)	82	2·03 (1·64, 3·42)
	**2009**	67	3·02 (1·61, 4·96)	67	1·73 (1·34, 2·23)
	**2010**	82	2·26 (1·80, 4·03)	81	2·00 (1·63, 2·62)
	**2011**	39	1·88 (1·21, 3·33)	36	3·14 (1·62, Undef)
**PET scan**	**Yes**	271	2·28 (2·00, 2·68)	270	1·88 (1·70, 2·18)
	**No**	179	1·64 (1·31, 2·06)	175	2·64 (2·23, 3·47)

Note: Undef = upper or lower limit of confidence interval is not defined.
Note: P-values for heterogeneity of treatment effect are 0·0004 for year of randomisation; and 0·0008 for use of PET scan.
Note: Use of PET scan by year of randomisation: 12% (2005), 18% (2006), 40% (2007), 77% (2008), 84% (2009), 90% (2010), 89% (2011).

Supplementary Figure S1 Disease-free survival

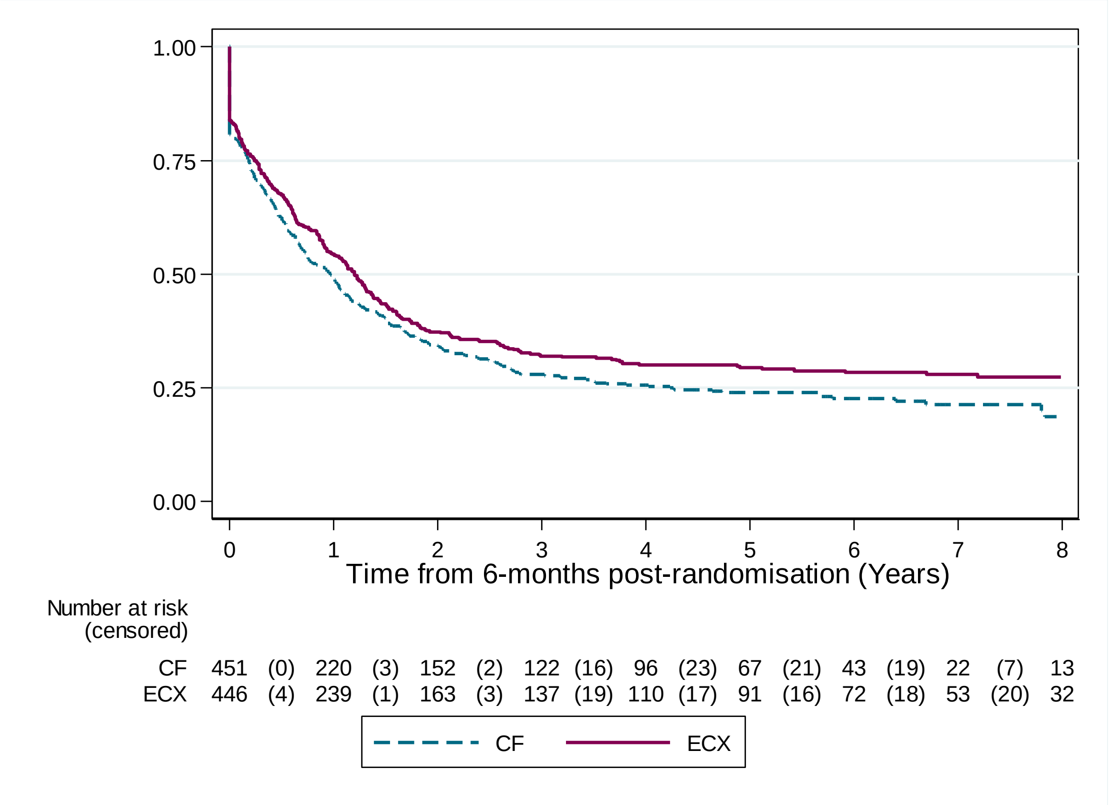

Supplementary Figure S2 Progression-free survival

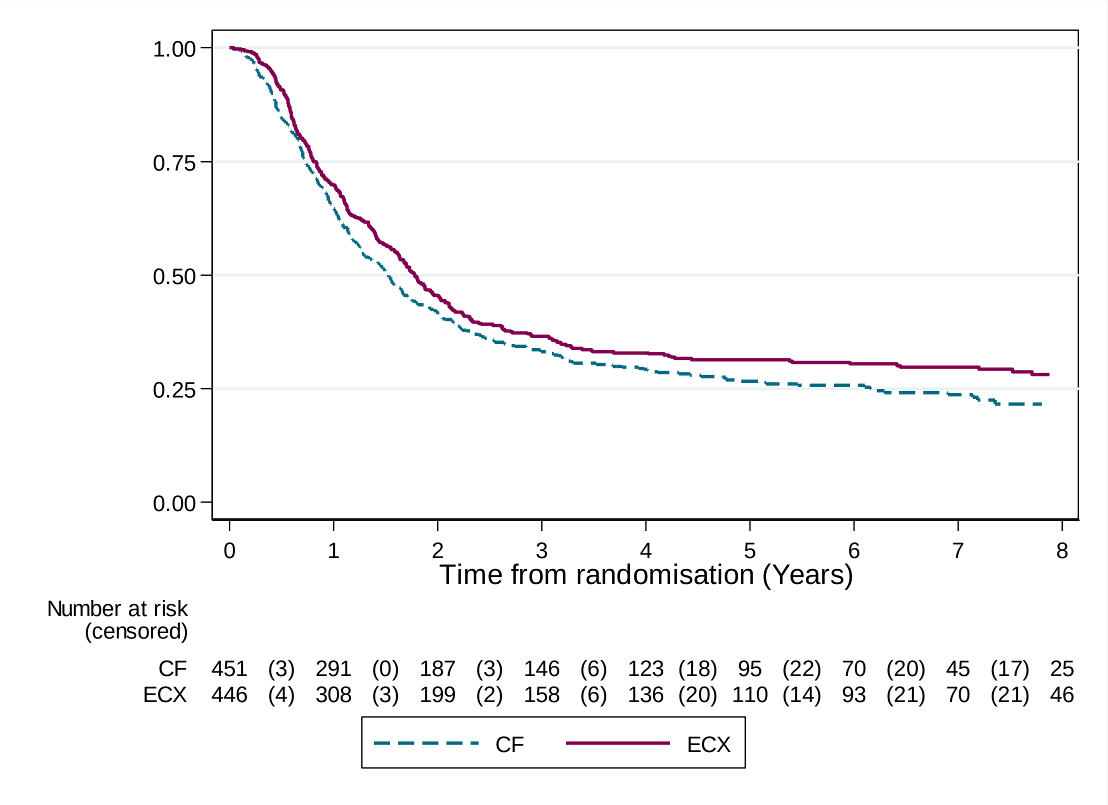

Supplementary Figure S3 Post-operative survival according to local Mandard TRG assessment.

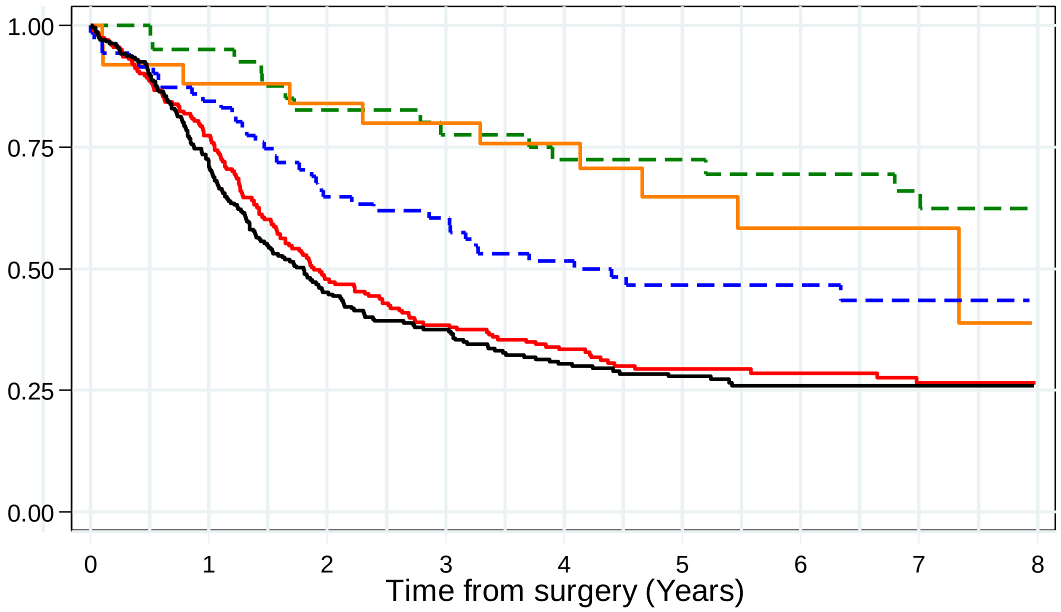

At risk (censored)																	
Grade 1	41	(0)	39	(1)	33	(0)	31	(2)	27	(2)	25	(2)	22	(3)	18	(8)	9
Grade 2	25	(0)	22	(0)	21	(1)	19	(2)	16	(3)	11	(1)	9	(3)	6	(5)	1
Grade 3	71	(0)	60	(1)	45	(1)	41	(1)	34	(6)	25	(7)	18	(3)	14	(6)	8
Grade 4	204	(1)	157	(0)	97	(0)	78	(6)	62	(12)	43	(9)	33	(8)	23	(7)	16
Grade 5	243	(2)	173	(2)	107	(2)	87	(8)	63	(9)	49	(12)	34	(12)	22	(6)	16

Note: HR (95% CI), p-value for comparison with grade 1 or 2 is 1.77 (1.06, 2.96), p=0.028 for grade 3, and 3.04 (2.01, 4.61), p<0.0001 for grade 4 or 5.
